# Resistin Modulates the Functional Activity of Colostral Macrophages from Mothers with Obesity and Diabetes

**DOI:** 10.3390/biomedicines10102332

**Published:** 2022-09-20

**Authors:** Letícia Damas Leão Dalcin, Danny Laura Gomes Fagundes-Triches, Adriele Ataides de Queiroz, André Henrique Furtado Torres, Danielle Cristina Honorio França, Tatiane Araújo Soares, Luana Cristina da Silva Ramos, Carla Roberta Silva Souza Antônio, Mahmi Fujimori, Eduardo Luzia França, Adenilda Cristina Honorio-França

**Affiliations:** Institute of Biological and Health Science, Federal University of Mato Grosso, Av. Valdon Varjão, 6390, Barra do Garças 78698-091, Mato Grosso, Brazil

**Keywords:** resistin, diabetes, obesity, cells, colostrum

## Abstract

Background: Obesity and diabetes are major public health problems. Resistin is an adipokine that links the two diseases. There are few reports regarding colostrum cells and resistin from mothers with obesity and diabetes. Thus, this study aimed to determine the functional activity of macrophages present in the breast milk and colostrum of diabetic mothers with obesity and the effects of resistin on these cells. Methods: The women were divided according to BMI and glycemic status into normal weight non-diabetic, obese non-diabetic, normal weight type 2 diabetic, or obese type 2 diabetic groups. ELISA determined the resistin in colostrum. The cell subsets and apoptosis were determined by flow cytometry and the functional activity of cells by fluorescence microscopy. Results: The resistin levels were higher in the colostrum from diabetic mothers with obesity. The frequencies of CD14^+^ cells and cells expressing CD95^+^, independent of resistin treatment, were higher in the colostrum from diabetic mothers with obesity. The frequency of cells expressing CD14^+^CD95^+^ was higher in cells not treated with resistin in the colostrum from diabetic mothers with obesity. Apoptosis, irrespective of the presence of resistin, increased, whereas microbicidal activity decreased in cells from diabetic mothers with obesity. Conclusion: The data suggest that hyperglycemia associated with low-grade inflammation caused by obesity affects the percentage of cells expressing CD14^+^CD95^+^, death by apoptosis, and microbicidal indices; meanwhile, resistin restored the microbicidal activity of colostrum cells.

## 1. Introduction

Diabetes mellitus is a metabolic disease characterized by hyperglycemia resulting from insufficient or absent insulin secretion from the pancreas, with or without concomitant impairment of insulin action, or both [1]. In pregnant women with type 2 diabetes or gestational diabetes mellitus (GDM), intrauterine hyperglycemic exposure of the fetus can lead to changes in neuroendocrine metabolism, influencing metabolic programming [2]. In women of reproductive age, the prevalence of diabetes has been reported to be 6.8%, with pre-gestational diabetes affecting 2% of all pregnancies [3]. Most women who have type 2 diabetes prior to conception are overweight or obese [4].

Like diabetes, obesity is a disease that affects the global population and pregnant women. It is characterized by the accumulation and storage of fat in the body, resulting from high food intake and reduced calorie expenditure [5]. It is known that adipose tissue consists of adipocytes capable of secreting numerous signaling cytokines, called adipokines [5]. Adipokines regulate multiple biological processes, such as inflammation, angiogenesis, fibrinolysis, vascular homeostasis, eating behavior, insulin sensitivity, and immunological processes [6]. 

The association between obesity and type 2 diabetes is well reported in the literature. About 3% to 15% of women develop diabetes mellitus during pregnancy [7]. Adipokine resistin is a link between diabetes and obesity. The human resistin pre-polypeptide precursor forms a 12.5 kDa molecule (108 amino acids) [8]. Resistin, produced by adipocytes and macrophages [9], belongs to the family of resistin-like molecules (RELMs), which are small secreted cysteine-rich proteins with hormone-like activity [10] that act in inflammatory processes [11,12].

This hormone secreted by adipose tissue resists the action of insulin and impairs glucose homeostasis, leading to the development of type 2 diabetes mellitus (DM2). In addition, human resistin is among the inflammatory regulators that act on macrophages [13], indicating that resistin plays a role in insulin resistance, obesity, and diabetes [14,15]. Resistin is also present in human milk and regulates a child’s growth, metabolic development, and appetite [16]. Although resistin plays an important role in the association of diabetes and obesity, little is known about the influence of this hormone on the immunological components present in human milk.

Human milk has adequate amounts of macro- and micronutrients and bioactive components that aid in the maturation of the immune system and development [17]. In obese mothers, despite the biochemical changes in their blood, these changes are not reflected in breast milk, reinforcing that this secretion provides immunological protection for newborns [18].

However, some studies suggest that maternal health status can directly interfere with the composition of breast milk and pathologies existing before the gestational period [18,19]. In addition, the immunological and hormonal patterns associated with changes in maternal metabolism may influence the development of a newborn’s immune system, contributing to greater susceptibility to infections via colostrum, which is a yellow viscous fluid rich in immunoglobulins, proteins, fat soluble vitamins, minerals and leukocytes, secreted 1–7 days postpartum [19]. The present study aims to determine the resistin levels of diabetic mothers with obesity and their effects on colostrum mononuclear cells.

## 2. Materials and Methods

### 2.1. Study Design and Participants

This cross-sectional study evaluated pregnant women (18–35 years old) recruited from the public health service in Barra do Garças, MT, Brazil. Before participating in the study, the volunteers signed an informed consent form, which was approved by the ethics committee. 

### 2.2. Subjects

In the first trimester of gestation, the women were divided into two groups according to their body mass index (BMI-[20]): the normal weight group, composed of those with BMI 18.5–24.9 kg/m^2^ (N = 28), and the obese group with BMI ≥ 30 kg/m^2^ (N = 27). Their blood glucose was analyzed to determine their glycemic level. The women underwent a 75 g oral glucose tolerance test (OGTT-75g – 1) applied between the 24th and 28th weeks of pregnancy. The OGTT-75g was positive when the plasma glucose values of postload 1 h of 180 mg/dL and postload 2 h of 153 mg/dL [21] were met, or they exceeded a fasting blood sugar level of 92 mg/dL. According to the OGTT-75g, 20 women were classified into the non-diabetic group (normal 75-g OGTT; N = 20). The women with type 2 diabetes mellitus (DM2) were referred to the Health Unit with a confirmed diagnosis (DM2; abnormal 75-g OGTT before pregnancy; N = 20). 

Glycemic control was assessed during pregnancy and was considered adequate when the mean blood sugar level was less than or equal to 120 mg/dl and inadequate when the mean blood sugar level was greater than 120 mg/dl. The glucose levels in maternal blood and colostrum were analyzed by the enzymatic system [18]. Patients with DM2 were treated with diet, exercise, and insulin therapy from the beginning of their pregnancy [21]. The non-diabetic pregnant women did not receive any intervention to control hyperglycemia. 

The inclusion criteria were as follows: (a) pre-gestational weight known or measured until the end of the 13th gestational week; (b) newborn gestational age at delivery between 37 and 41^6/7^ weeks; (c) negative serology for hepatitis, syphilis, and HIV, and (d) provided informed consent. Women with multiple pregnancies, DM1, GMD, fetal malformations, and deliveries before the 36^th^ week of gestation were excluded. 

### 2.3. Colostrum Sampling and Separation of Colostral Cells

Approximately 8 mL of colostrum from each woman was collected in sterile plastic tubes between 48 and 72 h postpartum. The samples were centrifuged (160× *g*, 4 °C) for 10 min, separating the colostrum by obtaining cell pellet and colostrum supernatant. The colostrum supernatant was stored at −80 °C for later examination of glucose and resistin levels. The cell separation was carried out using Ficoll Paque gradient (Pharmacia, Upsala, Sweden) and showed 96% mononuclear cells analyzed by light microscopy. Mononuclear cells were resuspended independently in serum-free medium 199 at a 2 × 10^6^ cells/mL final concentration. 

### 2.4. Resistin Levels Determination

The resistin concentration in the colostrum was determined using a Human Resistin ELISA kit (Immunoenzymatic Assay; Sigma, St. Louis, MO, USA). The assay range was inter-assay cv: <12%; intra-assay cv: <10%; sensitivity: 2 pg/mL. The reaction was measured by absorbance in a spectrophotometer with a 450nm filter. The data were calculated using a standard curve and are presented in ng/mL.

### 2.5. Resistin Treatment of Cells

Before analysis, the MN phagocytes (2 × 10^6^ cells/mL) were treated with 50 µL resistin (Sigma, St. Louis, MO, USA; 100 ng/mL) for 60 min at 37 °C. The phagocytes were then washed with Medium 199 at 4 °C and immediately used in the assays. A control was performed using only Medium 199.

### 2.6. Cell Subsets

Colostrum cells were stained with 5 μL of anti-CD4^+^ FITC and anti-CD3^+^ PerCP for 30 min at room temperature. In addition, the CD4^+^CD95 populations were also evaluated using anti-CD4+PerCP anti-CD95+PE (1.0 mg/mL). Cells were centrifuged in phosphate-buffered saline (PBS) containing bovine serum albumin (BSA; Sigma, St. Louis, MO, USA; 5mg/mL) and analyzed by flow cytometry. IgG1-FITC and IgG1-PE (BD Biosciences) were used as controls. Cells were analyzed by flow cytometer (FACSCalibur, BD Biosciences, Becton, Dickinson, San Jose, CA, USA) and data were obtained using the Flowjo 7.2.5 software (Becton, Dickinson, San Jose, CA, USA).

### 2.7. Apoptosis Assay

Firstly, in colostrum cells, apoptosis was detected by Hoechst 33342 (BD Bioscience, USA). Briefly, colostrum cells were fixed with methanol at −20°C for 10 min and stained with Hoechst for 60 min at 37 °C in the dark. The cells also were centrifuged and resuspended in BD Pharmingen^TM^ Stain Buffer and evaluated using a Flow Cytometer (FACS Calibur, BD Bioscience, USA).

Secondly, apoptosis was assessed using the APO-DIRECT™ kit (BD Biosciences—USA). The manufacturer’s instructions were used to obtain the data. The results were analyzed by flow cytometry (FACSCalibur system, Becton, Dickinson, San Jose, CA, USA) and the apoptosis index was obtained using Cell Quest software (BD Biosciences, USA).

### 2.8. Bactericidal Assay

We used enteropathogenic *Escherichia coli* (EPEC) to assess the functional activity of colostrum phagocytes. This bacterium was isolated from the stool of an infant with acute diarrhea (serotype 0111: H^-^ AL^-^, eae^+^, eaf^+^, bfp^+^). The EPEC was prepared and adjusted to 10^8^ bacteria/mL.

Phagocytosis and microbicidal activity were assessed using the acridine orange method. Equal volumes of EPEC and phagocyte suspension were incubated under continuous shaking at 37 °C for 30 min. Phagocytosis was stopped by incubation on ice, and the suspensions were centrifuged twice (160× *g*, 10 min, 4 °C). Cells were resuspended in Medium 199. The cells were stained for one minute with 200 μL acridine orange (14.4 g/L; Sigma-Aldrich), resuspended in Medium 199, washed twice, and were analyzed by fluorescence microscopy (400× and 1000× magnification).

The phagocytosis index was determined by the number of cells that ingested at least three bacteria in a pool of 100 cells. The microbicidal index was calculated as the ratio between orange-stained (dead) and green-stained (alive) bacteria ×100. All of the analyses were performed in duplicate.

### 2.9. Statistical Analysis

Analysis of variance (ANOVA) and Tukey’s post hoc test were used to evaluate the resistin, cell subsets, phagocytosis, microbicidal activity, and apoptosis index of colostrum MN cells treated with resistin or left untreated. Statistical significance was considered when *p* < 0.05.

## 3. Results

The clinical characteristics of the mothers are presented in Table 1. Colostrum glucose levels were higher, independent of BMI, in diabetic mothers (Table 1). The resistin concentration in the colostrum was higher in diabetic mothers with obesity (375.3 ± 120.2) than in normal weight non-diabetic mothers (141.1 ± 63.9). However, the resistin levels in the obese non-diabetic group (153.2 ± 69.7) and the normal weight DM2 group (181.7 ± 20.2) were similar to the respective samples from the normal weight non-diabetic group.

The frequency of cells expressing CD14^+^, independent of resistin treatment, was higher in the colostrum from diabetic mothers with obesity (43.8 ± 19.2 and 41.8 ± 19.1 with and without, respectively). The percentage of cells expressing CD95^+^ (38.4 ± 19.2 and 35.6 ± 9.1) was higher in the colostrum of the obese diabetic group treated or not treated with resistin (Table 2). The frequency of cells expressing CD14^+^CD95^+^ (45.8 ± 16.5) was higher in cells not treated with resistin in the colostrum from diabetic mothers with obesity. The resistin treatment reduced the percentage of cells expressing CD14^+^CD95^+^ (36.4 ± 9.2) in the colostrum from diabetic mothers with obesity (Table 2). 

**Figure 1:**
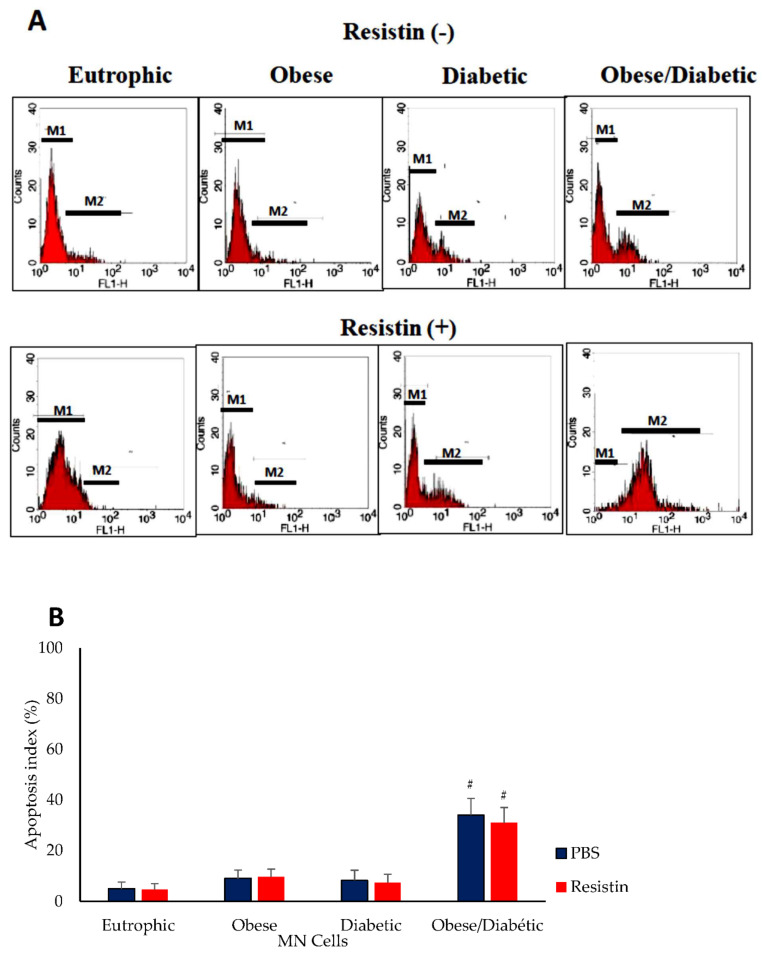
Apoptosis index (%) of colostrum mononuclear (MN) cells treated with resistin or left untreated from mothers with different glycemic and nutritional status. (**A**) Cells were labeled with fluorescein isothiocyanate deoxyuridine triphosphates (FITC-dUTP). The presence of apoptotic cells was demonstrated by increased fluorescence intensity (M2 gate). (**B**) Cell apoptosis index. Data were expressed as means and standard errors of eight MN cells from different individuals in each group (ANOVA, *p* < 0.05). ^#^ Significant difference between groups considering the same treatment.

Flow cytometry was performed to determine apoptosis induction in colostrum MN cells (Figure 1). There were increases in fluorescence intensity (M2 gate) in colostrum cells in the diabetic mothers with obesity (Figure 1A).

The apoptosis index of colostrum MN cells from the obese group (9.1 ± 1.3 without and 9.6 ± 1.0 with resistin) was similar to those cells from the diabetic group (8.3 ±1.3 without and 7.4 ± 1.3 with resistin) and there were no differences when compared with the colostrum cells from the normal weight group (4.9 ± 0.7 with and 4.7 ± 1.2 without resistin).

The colostrum cells from diabetic mothers with obesity, irrespective of the presence of resistin, presented an increase in the apoptosis index (34.0 ± 6.5 without and 30.9 ± 6.0 with resistin) when compared with the normal weight group (Figure 1B).

**Figure 2:**
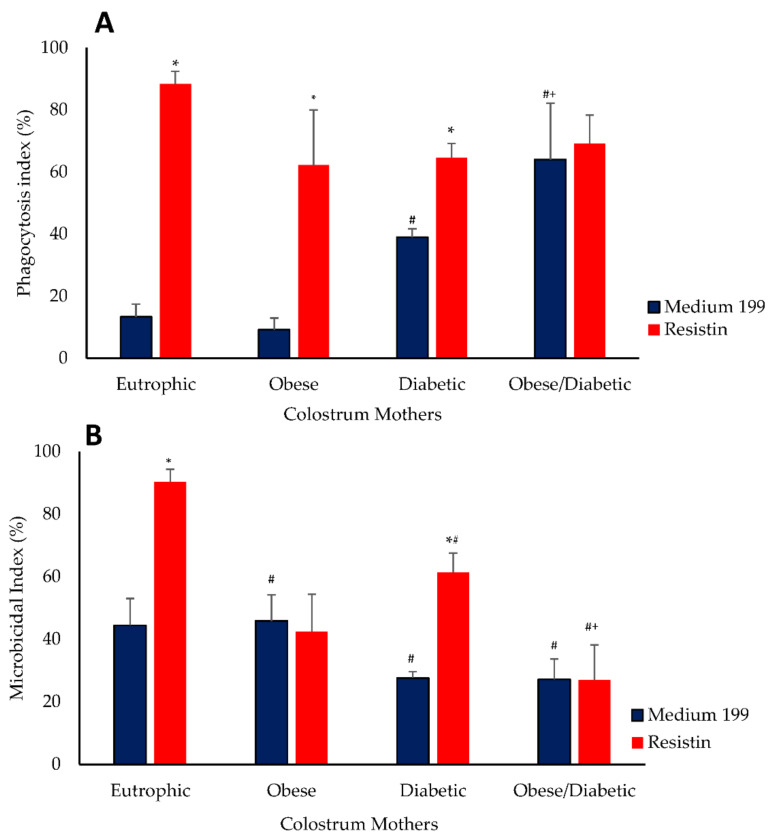
Phagocytosis (**A**) and microbicidal (**B**) activity of colostrum MN cells treated with resistin or left untreated from mothers with different glycemic and nutritional status. The data were expressed as means and standard errors of eight MN cells from different individuals in each group (ANOVA, *p* < 0.05). * Difference between treatments, considering the same group. # Difference between groups considering the same treatment. + Difference between diabetic and obese diabetic groups considering the same treatment.

The phagocytosis index was higher in the cells from mothers with diabetes and obesity (64 ± 8.2) when compared with cells from the diabetic group (39.0 ± 2.6), obese group (9.2 ± 3.7) and normal weight group (13.33 ± 4.0). The diabetic group showed a higher phagocytosis index when compared with the obese and normal weight groups. The resistin treatment increased the phagocytosis in colostrum cells in the normal weight group (88.33 ± 4.1), obese group (62.2 ± 7.8) and diabetic group (64.6 ± 4.5). Resitin treatment did not alter the phagocytosis index of colostrum cells from mothers with diabetes and obesity (without 64 ± 8.2 and 69.2 ± 9.2 with resistin). The highest phagocytic indices, after treatment, were observed in cells from normal weight non-diabetic mothers (Figure 2A).

The microbicidal activity was lower in the colostrum cells from the normal weight diabetic group (27.6 ± 2.0) and diabetic and obesity group (27.2 ± 6.5) when compared with cells from normal weight non-diabetic mothers (44.4 ± 8.6). The microbicidal activity was not altered in the colostrum cells from non-diabetic mothers with obesity (45.8 ± 8.3). The microbicidal activity of resistin-treated cells from normal weight and non-diabetic (90.4 ± 3.9) and normal weight and diabetic groups (61.4 ± 6.1) increased. The highest microbicidal index, after treatment, was observed in cells from normal weight non-diabetic mothers. Conversely, the lowest microbicidal indices were observed in colostrum cells (26.9 ± 9.2) from obese diabetic mothers (Figure 2).

## 4. Discussion

The maternal nutritional status before pregnancy can have consequences for the child. The passive immunity acquired through breastfeeding is essential for the newborn’s adaptation to the extrauterine environment [18,22]. The present study describes the functional activity of colostrum cells in the presence of resistin in hyperglycemic and obese mothers. Furthermore, we demonstrated that the association of hyperglycemia and obesity affects the apoptosis and microbicidal activity of colostrum MN cells.

Breast milk is rich in defense factors [23,24] which can be immunological supplements. In this work, the resistin was higher in the colostrum of diabetic mothers with obesity. Resistin is produced by adipocytes and macrophages [16], and it alters the action of insulin and impairs glucose homeostasis. In addition, resistin in breast milk is positively correlated with resistin in infant serum [23,24]. Thus, the increase in this adipokine in the colostrum reinforces the importance of controlling obesity and maternal blood glucose.

The infiltration of immune cells in adipose tissue has been demonstrated, of which 50% are macrophages [25]. As in the adipose tissue, in the human colostrum, macrophages constitute 40% of the secreted cells. Here, the colostrum of diabetic mothers with obesity showed an increase in the subsets of cells expressing CD14^+^. This molecule is a classic glycoprotein that can be found historically in the cell membrane [26,27], and is a marker of monocytes/macrophages [28]. These cells once activated release sCD14 [29]. This molecule in its form is found circulating in large quantities in patients with sepsis [30] and in inflammatory processes [31]. CD14 is a molecule that also regulates the production of pro-inflammatory cytokines and lipid mediators [32,33]. In this study, this increase was independent of the treatment of cells with resistin, suggesting that the greater percentage of these cells results from the maternal hyperglycemic and inflammatory state.

The inflammatory response due to obesity is associated with diabetes pathophysiology [14] and has been used to explain the relationship between diabetes and obesity. Hyperglycemia can alter Treg cell and Fas receptor (CD95^+^) expression in T cells. In this study, the percentage of cells expressing CD95^+^, regardless of resistin, was higher in the colostrum of obese diabetic mothers. The frequency of CD14^+^ cells expressing CD95 (Fas) increased in the colostrum cells of obese diabetic mothers only when they were not previously stimulated by resistin. Fas is a cell surface receptor that plays a central role in regulating the death of many cell types, including pancreatic β cells, and may be associated with the development of type 2 diabetes [34]. CD95 (Fas) also acts on chronic inflammation by inducing non-apoptotic signaling pathways [35,36]. CD95 stimulation can trigger apoptosis or activate pro-inflammatory signaling pathways. This receptor has also been described as an activator of pattern recognition receptors (PRRs) known to detect damage-associated molecular patterns (DAMPs), such as DNA debris and dead cells, and thus may contribute to the pro-inflammatory role of CD95 [37].

We observed an increase in the expression of CD95^+^ and apoptosis in colostrum cells from obese diabetic mothers. A possible explanation for higher apoptotic cell indices may be the association of hyperglycemia and low-grade inflammation, since exogenous resistin did not change the percentage of cells expressing CD95^+^ or the apoptosis. Insulin alterations should also be considered, as this hormone plays an important role in the Fas pathway [38] and has been linked to insulin resistance and diabetes [39]. Higher Fas expression in blood, lower insulin sensitivity, and adipose tissue dysfunction have been observed in diabetic and obese patients [40]. 

Obesity and diabetes are related to a higher incidence and severity of infectious diseases and involve different cells and immune mechanisms [41]. In addition, the immunological factors observed cause alterations in the percentage and function of cell, cytokine, antibody, and adipokine levels [18,42,43], suggesting changes in inflammatory mediators [44]. 

In this study, the increase in cells expressing CD14^+^CD95^+^ and apoptosis due to hyperglycemia and low-grade inflammation could alter the functional activity of colostrum macrophages. There was an increase in phagocytosis, both in the colostrum of normal weight diabetic mothers and diabetic mothers with obesity. This increase may be related to the inflammatory processes characteristic of diabetes and obesity. However, the high phagocytic indices did not reflect the microbicidal capacity of these cells. On the other hand, treating cells with resistin restored the microbicidal activity of colostrum macrophages from diabetic mothers but not from obese diabetic mothers.

In diabetic patients, resistin appears to be linked to insulin resistance [45] and has played a role in upregulating pro-inflammatory cytokines [14]. Breast milk controls a child’s metabolic development [46]. In blood, resistin also appears to be present in stressful situations. Resistin accumulation prevents cellular stress and contributes to the cell going into apoptosis [9]. Here, the increased death by apoptosis in CD14^+^ cells expressing CD95^+^ from the colostrum of diabetic mothers with obesity influenced the functional activity of these cells, reducing their microbicidal capacity.

A limitation of this study that should be considered is that these data were evaluated in one period of collection and only one milk maturation stage. It is necessary to continue investigations focusing on other factors that may be involved during breastfeeding in mothers with diabetes and BMI alterations.

## 5. Conclusions

The results from this study suggest that hyperglycemia associated with low-grade inflammation due to obesity alters the percentage of cells expressing CD14+CD95+, death by apoptosis, and microbicidal indices. Furthermore, resistin restored the microbicidal activity of colostrum cells from diabetic mothers but not from mothers with comorbidities, suggesting a possible effect of this hormone on the colostrum of mothers with hyperglycemia. Thus, controlling maternal obesity-associated resistin may be another way to regulate the functional activity of colostrum macrophages. Finally, these data reinforce the hypothesis that breastfeeding is important for maternal and child health, especially for hyperglycemic and obese mothers.

## Figures and Tables

**Table 1:** Clinical data of mothers with different glycemic and nutritional status.

Parameters	Normal Weight Non-Diabetic (N = 28)	Obese Non-Diabetic (N = 27)	Normal Weight Diabetic (N = 20)	Obese Diabetic (N = 20)
Age (years)	28.3 ± 4.1	27.4 ± 3.9	32.3 ± 6.2	31.5 ± 4.3
Gestational age (weeks)	38.9 ± 2.5	37.2 ± 3.5	37.5 ± 1.4	36.8 ± 4.5
BMI-1	23.7 ± 2.1	32.8 ± 4.6	24.2 ± 1.6	34.8 ± 5.2
BMI-2	33.6 ± 4.2	39.2 ± 4.9	36.4 ± 4.8	41.1 ± 4.5
Hypertension (%)	0	10	15	25
Physical exercise (%)	38	35	65	75
Blood glucose level (mg/dL)	85.5 ± 4.6	92 ± 5.7	114.7 ± 7.5 *	119.8 ± 6.8 *
Colostrum glucose level (mg/dL)	76,8 ± 5.5	81.2 ± 0.44	101.4 ± 4.1 *	112.4 ± 3.9 *
Resistin (ng/mL)	141.1 ± 63.9	153.2 ± 69.7	181.7 ± 20.2	375.3 ± 120.2 *

BMI-1: body mass index in the first trimester of pregnancy; BMI-2: body mass index in the third trimester of pregnancy (ANOVA, *p* < 0.05). * Significant differences between treatment groups (obese non-diabetics, normal weight diabetics, obese diabetics) and the normal weight group.

**Table 2:** Percentage of CD14^+^ cells treated with resistin or untreated, expressing Fas (CD95) in mothers with different glycemic and nutritional status.

(%) Cells Expressing	Resistin	Normal Weight Non-Diabetic	Obese Non-Diabetic	Normal Weight Diabetic	Obese Diabetic
	No	29.1 ± 14.5	37.1± 10.3	34.6 ± 10.9	43.8 ± 19.2 *
CD14^+^	Yes	28.6 ± 12.2	29.5 ± 11.8	30.5 ± 7.5	41.8 ± 19.1 *
	No	21.8 ± 14.5	31.4± 10.3	27.0.6 ± 10.9	38.4 ± 19.2 *
CD95^+^	Yes	23.6 ± 5.2	25.6 ± 10.4	27.2 ± 8.3	35.6 ± 9.1 *
	No	21.3 ± 12.2	30.0± 12.1	25.9 ± 9.4	45.8 ± 16.5 *
CD14 ^+^ CD95^+^	Yes	22.7 ± 4.1	24.4 ± 10.2	24.1 ± 7.2	36.4 ± 9.2 *^#^

Data are expressed as means and standard errors of 10 MN cells from different individuals in each group. (ANOVA, *p* < 0.05). * Indicates intergroup differences, considering the same treatment. ^#^ Indicates treatment differences, considering the same group.

## Data Availability

The data that supported the interpretations of this study will be made available by the authors if requested.

## Abbreviations

ANOVA	Analysis of variance
BMI	Body mass index
CD14^+^	Cluster of differentiation 14
CD95^+^	Cluster of differentiation 95—Fas receptor
DAMPs	Damage-associated molecular patterns
DM2	Type 2 diabetes mellitus
dUTP	Deoxyuridine triphosphates
EPEC	Enteropathogenic *Escherichia coli*
FITC	Fluorescein-5-isothiocyanate
GDM	Gestational diabetes mellitus
MN cells	Mononuclear cells
OGTT	Oral glucose tolerance test
PE	Phycoerythrin
RELMs	Resistin-like molecules
sCD14	Soluble cluster of differentiation 14
